# Early presentation of clinical hereditary angioedema symptoms in an infant

**DOI:** 10.1186/1710-1492-10-S2-A36

**Published:** 2014-12-18

**Authors:** Hoang Pham, Stephanie Santucci, William H Yang

**Affiliations:** 1 University of Ottawa Medical School, Ottawa, ON, Canada; 2 Allergy and Asthma Research Centre, Ottawa, ON, Canada

## Background

Timely diagnosis of hereditary angioedema (HAE) is challenging in children. The barriers include lack of awareness of HAE, communication difficulties, diagnostic testing limitations, and broad differential diagnoses for symptoms of HAE. Consequently, there has been no definitive study on the age of onset of symptoms of HAE in children. This lack of awareness can result in reduced quality of life due to suboptimal treatment of symptoms, significant delay in diagnosis, and/or misdiagnosis, which can result in unnecessary tests, treatments, and procedures. Current literature suggests that the mean age of onset is in the second decade of life, which is worsened by puberty, estrogen containing contraception, or estrogen hormone replacement therapy, but symptoms can also be present under one year. Here we present a case report of an infant not previously diagnosed with clinical symptoms of HAE but born from a mother with type I HAE.

## Method

Cord blood for C1-INH and C4 protein quantities were obtained at time of delivery. Parents were requested to monitor the child for symptoms and pictures were taken to document any clinically suspicious edema and/or rashes. Repeat laboratory testing was done after 1 year of age.

## Results

Cord blood results show C1-INH <0.12 (0.21-0.39) g/L and C4 0.08 (0.07 – 0.30) g/L. At 9 months, the child’s mother noted slight periorbital edema, which was documented with pictures. At 14 and 18 months, the child developed a rash on her torso and arms that resembled erythema marginatum; both episodes were documented with pictures.

## Conclusion

Clinical symptoms of HAE may begin as early as 9 months without any triggers. Parents and clinicians need to be vigilant and properly diagnose HAE to optimize the quality of life for these young patients and their families. We emphasize a high index of clinical suspicion of HAE should begin even in the early infant period.

